# Toward a Comprehensive Domestic Dirt Dataset Curation for Cleaning Auditing Applications

**DOI:** 10.3390/s22145201

**Published:** 2022-07-12

**Authors:** Thejus Pathmakumar, Mohan Rajesh Elara, Shreenhithy V Soundararajan, Balakrishnan Ramalingam

**Affiliations:** Engineering Product Development, Singapore University of Technology and Design, Singapore 487372, Singapore; pathmakumar_thejus@mymail.sutd.edu.sg (T.P.); rajeshelara@sutd.edu.sg (M.R.E.); shreenhithy@sutd.edu.sg (S.V.S.)

**Keywords:** domestic dirt, dirt dataset, audit robot, cleaning benchmark, dirt classification, robot-aided cleaning auditing

## Abstract

Cleaning is an important task that is practiced in every domain and has prime importance. The significance of cleaning has led to several newfangled technologies in the domestic and professional cleaning domain. However, strategies for auditing the cleanliness delivered by the various cleaning methods remain manual and often ignored. This work presents a novel domestic dirt image dataset for cleaning auditing application including AI-based dirt analysis and robot-assisted cleaning inspection. One of the significant challenges in an AI-based robot-aided cleaning auditing is the absence of a comprehensive dataset for dirt analysis. We bridge this gap by identifying nine classes of commonly occurring domestic dirt and a labeled dataset consisting of 3000 microscope dirt images curated from a semi-indoor environment. The dirt dataset gathered using the adhesive dirt lifting method can enhance the current dirt sensing and dirt composition estimation for cleaning auditing. The dataset’s quality is analyzed by AI-based dirt analysis and a robot-aided cleaning auditing task using six standard classification models. The models trained with the dirt dataset were capable of yielding a classification accuracy above 90% in the offline dirt analysis experiment and 82% in real-time test results.

## 1. Introduction

Cleaning is an inevitable routine associated with every domain. According to the recent market studies, the professional cleaning industry is steeply growing and expected to reach a market size of USD 88.9 billion by 2025 [1,2]. The growth of the cleaning industry is further boosted up by the increasing demand during the pandemic outbreak. A plethora of leading edge technologies have been introduced to the field of domestic and professional cleaning to enhance the performance of cleaning and maximize the productivity for the past decade [3,4,5]. This includes the usage of novel cleaning strategies using floor cleaning robots [3], Ultra-Violet-C (UVC) disinfection robots [4], cable-driven wall cleaning robots [5], etc. Currently, the reported studies about the cleaning performance enhancement are centered on the development of novel classes of cleaning robots and its associated components including robot efficient navigation, control, multi-robot cooperation, etc. For example, a morphology switching strategy for maximizing the area coverage in reconfigurable cleaning robots is reported [6]. Fuzzy inference systems used for enhanced adhesion awareness in vertical glass wall cleaning robots are reported [7]. An adaptive floor cleaning strategy by analyzing the human density is detailed in [8]. The research work mentioned in [9] presents an efficient charging mechanism for cleaning robots using infrared spots and neural network-based location estimators. A novel functional footprint-based efficient ship hull cleaning method using evolutionary algorithms is reported in [10]. The preceding analysis shows that a significant amount of research has focused on adapting the latest technologies for enhancing the performance of cleaning. However, a systematic method for assessing the quality of cleaning delivered by various cleaning methods has not been studied predominantly.

Dirt analysis is one of the critical elements for cleanliness auditing. The major challenges in the field of dirt analysis targeting cleaning auditing include the following:

- Even though there are proven AI models that could yield superior accuracy, there is no comprehensive dataset that the experts can use to train the AI models for dirt analysis.
- The dirt particles are often detected by typical computer vision-based analysis. However, capturing the finer features of the dirt is essential for AI-based analysis.
- The domestic dirt has identical visual features, which makes classification challenging for the AI models, which demands a high-quality dataset highlighting the visually distinct features of the dirt particles.

This research work presents a novel domestic dirt image dataset for cleaning auditing application including AI-based dirt analysis and robot-assisted cleaning inspection. Unlike the conventional vision-based dirt detection algorithms, whose application is limited to cleaning robots, the proposed method presents a dirt detection and classification strategy for cleaning auditing, where an audit sensor gathers the dirt samples via adhesive dirt lifting. As part of this work, a comprehensive dataset consisting of microscope images of commonly occurring domestic dirt is acquired using the sample audit sensor. To distinguish the features, each dirt image is analyzed in under 10× magnification. In addition, the selected dataset’s usability is further analyzed using training and validation accuracy in different deep-learning architectures that enable deep-learning-based dirt analysis. To the best of the author’s knowledge, a dataset of microscopic domestic dirt images has not been reported so far. The proposed research work opens the door toward new fronts for AI-driven dirt analysis targeting the domain of cleaning auditing. The The general objective of this research work is subdivided into the following:

1. Gathering of magnified images of domestic dirt particles using adhesive dirt lifting;
2. Analyze the usability of an acquired dataset in training and classifying the domestic dirt in standard classification models, using a cross-validation technique;
3. Analyze the performance of the proposed scheme in a real-time scenario by rolling out the trained classification model for real-time dirt composition estimation for an in-house developed audit robot.

The rest of the article is structured as follows. Section 2 provides a detailed study on the related works, Section 3 provides a birds-eye view of the adopted methods and methodology; Section 4 reports a detailed description of our analysis and experiments conducted in this research effort followed by Section 6, which concludes our findings.

## 2. Related Works

Despite the importance, cleaning auditing is analyzed in very few domains. For example, the formation of tests for cleaning quality analysis in the fish processing industry is reported in [11]. Lewis et al. proposed a modified adenosine triphosphate (ATP) benchmark for estimating the quality of cleaning for hospital environments [12]. Efforts toward establishing cleaning standards for the hospital environment are reported [13]. Similarly, Aziz et al. proposed microbiological monitoring for cleaning analysis targeting hospital contamination estimation [14]. The state-of-the-art cleaning auditing methods are centered around microbiological analysis and the ATP bioluminescence method [15]. However, the analysis method mentioned above is not scalable to most domains, including professional and domestic floor cleaning. The pioneering effort in cleaning auditing is proposed by Pathmakumar et al., where cleaning auditing is done with the help of a autonomous mobile robot and a sample audit sensor [16]. The research work mentioned above uses a cleaning auditing sensor that extracts the dirt from the floor by adhesive dust lifting followed by analysis using computer vision-based techniques. The auditing of a larger area is achieved with the audit robot using dirt exploration methods [17]. The robot-aided cleaning auditing is a viable approach for post-cleaning analysis compared to the laborious microbiological methods, which are limited only to a specific domain. The robot-aided cleaning auditing executes the cleaning auditing in a fully automated fashion, which bridges the lack of proper post-cleaning analysis in the domain of automated cleaning. One of the main challenges in robot-aided cleaning auditing is the development of an effective method for dirt analysis. Conventionally, visual detection methods are often used for dirt identification. For instance, Grunauer et al. proposed an unsupervised dirt spot detection where the problem is addressed as a binary classification problem [18]. Similarly, Bormann et al. proposed a training-free dirt detection framework in an office environment [19]. The above-mentioned method is further improved by a multi-class machine learning-based dirt detection method using a modified YOLOv3 framework [20]. A similar approach is reported where a cascaded neural network is used for multi-class debris detection for floor cleaning robots [21]. The current dirt detection algorithms are reported in [18,19,20,21]; here, the dirt detection is targeted at a cleaning robot to perform a selective cleaning. However, for the concerned scenario of cleaning auditing, a microscopic analysis of domestic dirt sampled after a dust-lifting process is inevitable. However, the present dirt detection frameworks are not designed for the above-mentioned application.

## 3. Methodology

**Figure 1:**
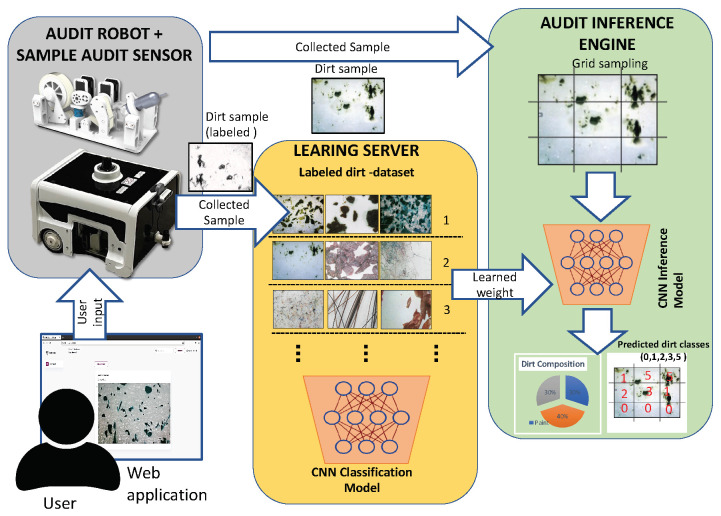
Proposed overview for domestic dirt collection using autonomous robot.

This work adopts our in-house developed cleaning auditing robot (BELUGA) for dirt dataset collection. In order to classify the domestic dirt, it is essential to build a comprehensive dataset of domestic dirt. The domestic dirt is referred to as particulate contaminates (usually measured in microns) that are carried by the airflow and settled down in undisturbed air. The samples are collected from indoor and semi-indoor regions by attaching the sensors to the BELUGA robot. The overview of the sample collection procedures and method for dirt inference is depicted in Figure 1. The sample collection procedure involves the gathering of different sets of dirt samples from floor surfaces. The samples can be collected either by using the sample audit sensor in a standalone way or by using an autonomous robot carrying a auditing sensor payload. The sample audit sensor gather images using the adhesive dirt lifting principle, which is an established principle used for forensic trace collection [22,23]. For the analysis part, the images of the adhesive tape are taken using a microscopic camera with 10× magnification. The images gathered using the sample audit sensor are stored in a remote database on a cloud server. In case of limited connectivity to the remote server, the captured images are enhanced and stored locally onboard the robot. The sample collection is controlled and monitored using a web application hosted on the local device that serves as an interface for the user to control the process. In the remote server, the images are stored in a labeled manner.

### 3.1. Robot Architecture

**Figure 2:**
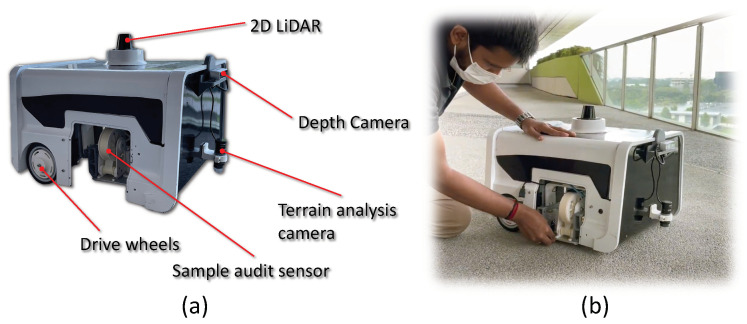
Cleaning audit robot—BELUGA (**a**); Attaching of sample audit sensor with BELUGA (**b**).

**Figure 3:**
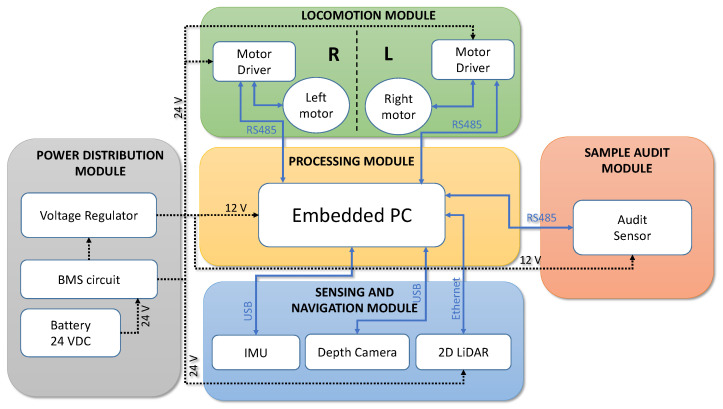
The system architecture of the BELUGA robot with the main subsystems.

The audit robot is the carrier of a sample audit sensor, and it helps perform the dirt sample gathering from a vast area of space where the manual sample gathering is not feasible. The audit robot called BELUGA is an in-house developed autonomous cleaning audit robot integrated with the sample audit sensor. The Figure 2 shows BELUGA robot and mounting of sample audit sensor onto the robot. The BELUGA robot is comprised of a locomotion module, power distribution module, navigation module, audit sensor and processing module (shown in Figure 3).

The locomotion unit of the robot is composed of a pair of brushless DC (BLDC) motors that form a differential drive wheel configuration. The third point of contact of the robot is made using a free rotating castor wheel. The BLDC motor drivers established a closed-loop velocity control for the driving wheels. The velocity feedback is obtained from the BLDC motor drivers using the incremental encoders associated with the motor. The velocity feedback from the motors is used for computing the odometry information. The communication with the drive motors is established using MODBUS-RTU communication. The robot uses a 24 V DC lithium-ion phosphate battery to power all subsystems. The main supply from the battery is regulated and distributed further to power-sensitive components. A 2D LiDAR is the primary perception sensor associated with the robot, which is supported by a depth camera to add 3D perception capabilities and detect obstacles below the level of LiDAR. The LiDAR used in the robot is SICK TIM 581 with a range of 20 m in semi-outdoor conditions. The Intel Real-sense D435i is the depth camera with a resolution of 640×480 and $87^{\circ }\times 58^{\circ }$ angular field of view. Using Kalman filter-based state-estimation techniques, the wheel odometry information is generated by fusing the wheel velocity feedback from the motor drivers and the inertial measurement unit (IMU). The IMU used in the BELUGA robot is VectorNav VN100, which is a nine-axis IMU with built-in noise filtration.

The robot performs autonomous navigation in real time using the input from the perception sensors and odometry information. The robot possesses an embedded computer with an Intel core i7 processor and runs with an operating system of Ubuntu 20.04. The perception and navigation algorithms run alongside the ROS middleware in the embedded computer. Apart from the navigation sensors, the robot is integrated with the sample audit sensor. The communication between the sample audit sensor and the embedded PC is established through USB and RS485 communication. The USB established a communication link with the digital microscope, and RS485 is used to control the servo motors’ actuation inside the sensor. On the BELUGA robot, the sensor is attached to a removable sensor bay, which allows detaching the sensor from the robot.

### 3.2. Sample Audit Sensor

**Figure 4:**
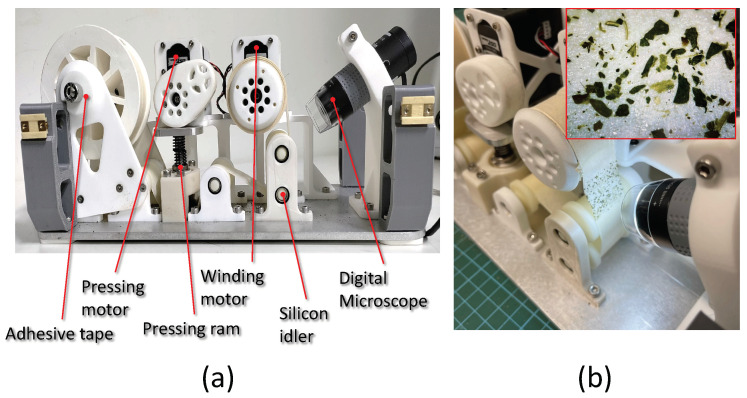
The sample audit sensor with major components (**a**) and the extracted dirt particles under the view of the microscope (the 10× magnified image captured in the inset) (**b**).

The major components associated with the sample audit sensor are the adhesive tape, winding motor, pressing motor, pressing ramp, and a digital microscope. The gathering of the dirt samples is done by the synchronized motion of winding motors and pressing motors. The winding motors and pressing motors are digital servos that can be position-controlled according to the data passed over the RS485 communication protocol. The winding motor of the sensor rotates an adhesive tape, and the pressing motor actuates the pressing ram that exerts downward force to press and release the sticking surface of the tape against the surface. The winding motor displaces the tape surface toward the field of view of the digital microscope to capture the magnified image of the surface of the tape. The pressing of the adhesive tape is completed for a 2 cm × 2 cm area; hence, analysis of the dirt is localized to the same surface area. Every sampling completed by the BELUGA robot takes 13 to 16 s, which includes 4 s to lower the sensor bay to the floor, 2 s to conduct a pre-sampling winding, 4 s for stamping action, and 3 to 6 s to perform a post-sampling winding. The post-sampling winding varies from 3 to 6 s since the length to displace varies as the adhesive tape radius decreases linearly as more and more samples are taken. The movement of the adhesive tape is guided through silicon idlers so that the adhesive tape will not become stuck while displacing the stamped area to the field of view of the microscope. Figure 4 shows the sample collection sensor module. The sensor consists of the (a) collection mechanism to gather dirt from the floor and pass it to the (b) microscope camera to capture, transfer and process the sample images.

## 4. Experiments and Analysis

This section describes the experimental and analysis procedure of dirt dataset collection and evaluation methods. The experiment has been performed in four phases. The first phase involves dataset curation. The second phase validates the collected dirt dataset through a k-fold cross-validation scheme and converging time of the various image classification algorithm. The third phase involves validation of the dirt dataset through the AI-based dirt classifier algorithm. The final phase involves validating the trained dirt classification models with the BELUGA robot on a real-time field trail.

### 4.1. Dataset Curation

**Figure 5:**
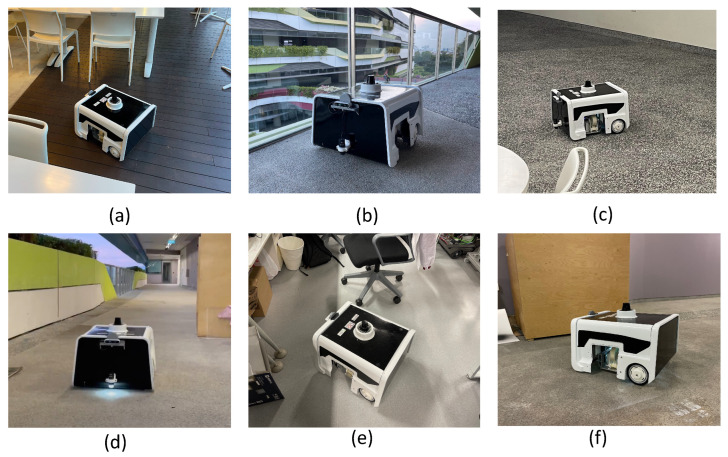
The dirt sample collection using the BELUGA robot in different locations such as a food court (**a**), semi-indoor walkway (**b**), office pantry (**c**), long corridor (**d**), office space (**e**), and warehouse (**f**).

The BELUGA robot is set to exploration mode, where it explores the region using the frontier exploration method, and the sample gathering is completed every 10 s. Figure 5 shows sample collection performed at different locations using the BELUGA robot. We selected a food court (Figure 5a), semi-indoor walkway (Figure 5b), office pantry (Figure 5c), long corridor (Figure 5d), office space (Figure 5e) and warehouse (Figure 5f) as the sites for data collection. Once the robot explores 98% of the deployed area, the robot resets its map and completes the frontier exploration again.

### 4.2. Dirt Class Identification

The sample collection procedure is repeated for 2 days in each location, and the composition of dirt is analyzed. The main dust particles lifted by the adhesive tape include ashes, hair (mostly from walkways and corridors), tiny bits of paper, sand, soil, etc. The bits of paper are collected from all the six locations taken for the data collection. The traces of sand and soil particles were also captured from all locations—however, the sand and soil particle concentration was higher in the walkways and corridors. The traces of food particles were also identified from every location; however, the most frequent occurrence of food particles in the collected samples was from the food court and pantry. Paint particles are captured in the sample collection completed in warehouses often, and it is observed that more traces are captured when the robot completes sampling near the corners and walls of the warehouse. Traces of seeds, lint, etc. were also identified from the collected samples across all locations. From the observations made from the dirt sample collection, the specified classes for the dirt data includes ash, hair, sand, soil, paper, paint, food, and fiber. These classes are finalized for the dirt dataset considering the following:

- Frequent occurrence during sample collection;
- Presence in every location;
- The health and environmental factors associated with identified dirt particles [24,25,26].

The identified dirt classes for the dataset are provided in the Table 1.

### 4.3. Dataset Preparation and Training

Upon identifying the distinct dirt classes, the image captured by the microscope at a resolution of 1280×720 was decimated to 16 image samples, each of size 320×120 images. The dirt samples collected by the robot as well as the samples manually collected were labeled and formed the dataset for domestic dirt. The samples where the dirt is spread less than 60% in the image area are discarded. Images overlapping with different classes are also discarded. After discarding the invalid images, a dataset of 3000 samples from each class is curated. From every class of labels images, 2500 images are taken for training and cross-validation, and the remaining 500 are used for offline testing.

### 4.4. Dirt Dataset Validation

The quality of the dataset is analyzed by inspecting the accuracy of the state-of-the-art AI-based image classification models trained using it. The quality of the collected dirt dataset was evaluated through training accuracy with a k-fold cross-validation scheme and statistical measure. For analysis, we choose two sets of classification models. The first set is composed of three less dense neural network architectures, and the second set is composed of three dense-layer neural networks. For the less dense models, we took VGG-11 [27,28], VGG-16 [27] and MobileNetV2 [29]. For the second set, for the dense-layer models, we choose ResNet50 [30,31], ResNet101 [30] and Darknet53 [32].

#### 4.4.1. K-Fold Cross-Validation Method

The k-fold cross-validation is completed by following the procedures given below:

1. Select the fold k=5.
2. Split the dataset to *k* groups, which are also known as folds.
3. Select k−1 folds for training the model and one fold for testing.
4. For every iteration, a new model is trained independent of the previous iteration.
5. Repeat the training and cross-validation *k* times; in every iteration, the remaining fold will serve as the test set.
6. The accuracy is determined on the kth iteration as the average of all iterations.

The six-image classifier model was trained with an early stopping condition to avoid the over-fitting of the model. The models are trained in NVIDIA GeForce 3080 GPU with a batch size of 32 and a learning rate of $4\times 10^{-3}$. Cross-entropy loss is used to estimate the model’s prediction performance in every forward pass [33]. Comparing the accuracy of every iteration provides insight into the curated dataset’s reliability and trustworthiness. However, a k-fold gives a more stable and trustworthy result since training and testing are performed on multiple combinations of test–train set decimation from the dataset.

**Figure 6:**
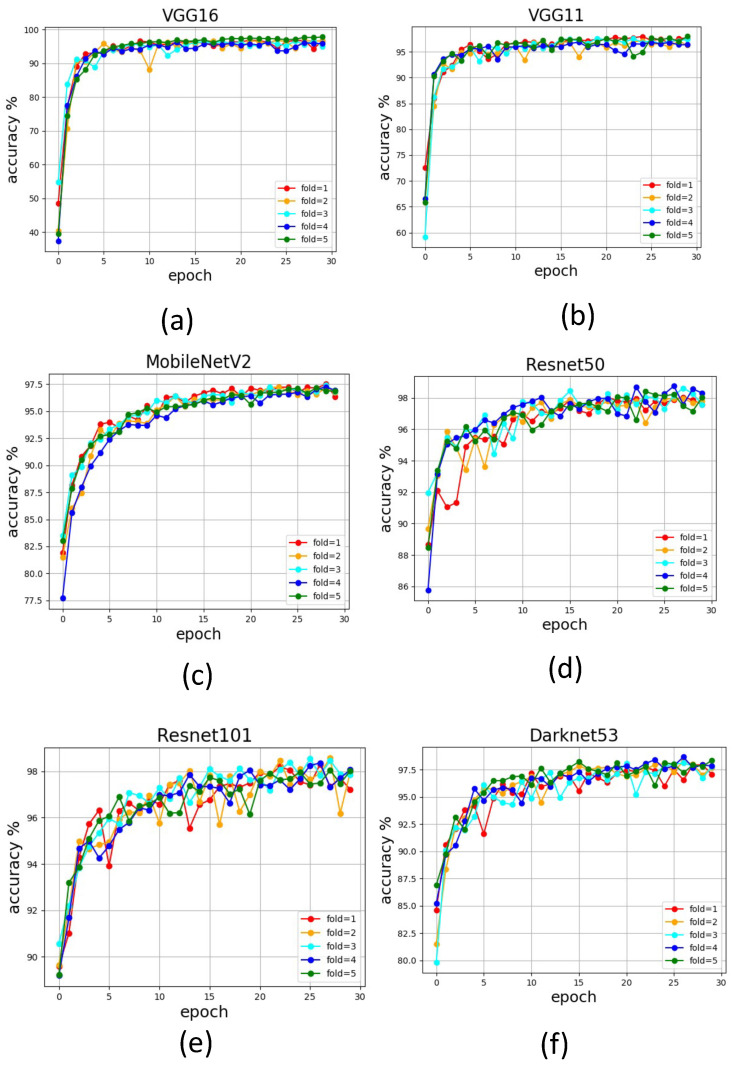
The accuracy profile for different classification models trained using curated dirt dataset; VGG-16 (**a**), VGG-11 (**b**), MobileNetV2 (**c**), Resnet50 (**d**), Resnet101 (**e**), Darknet53 (**f**).

Figure 6 reports the convergence profile for the models for the first 30 epochs of training. All models converged above 90% accuracy in every fold for all the six models. The average accuracy of every fold of training in VGG-11, VGG-16, and MobleNetV2 is observed in the first 10 epochs of training. A slightly delayed convergence is observed for dense-layer models such as ResNet50, ResNet101, and Darknet53. The dense-layer models took 15–20 epochs to see a consistency in the convergence trend. This trend is attributed to the relatively complex nature of ResNet50, ResNet101, and Darknet53 compared to the other models. One of the key indicators that is attributed to the quality of the dataset is the deviation of accuracy in every fold of training. The average training accuracy for k-fold and standard deviation in accuracy is tabulated in Table 2. All models show a small standard deviation in the k-fold training, which indicates a non-biased and balanced dataset. The variation in accuracy in different folds of training is comparatively less when it comes to dense-layer models. The minimal deviation is reported by ResNet101 (1.89), and maximum accuracy in training is reported by ResNet50 (96.58%).

#### 4.4.2. Dirt Dataset Validation through Statistical Measure

The dirt dataset’s efficiency was validated through a statistical measure function. Here, the models trained using our dirt dataset classification accuracy were chosen as the evaluation matrix to assess the dirt dataset quality. Accuracy Equation (1), precision Equation (2), recall Equation (3) and $F_{measure}$ Equation (4) were used to evaluate the trained model’s classification accuracy. The confusion matrix function was used to find the variables tp (true positives), fp (false positives), tn (true negatives) and fn (false negatives) through which accuracy, precision, recall and $F_{measure}$ were calculated. The evaluation metrics includes:

(1) $$Accuracy\left(Acc\right)=\frac{tp+tn}{tp+fp+tn+fn}$$

(2) $$Precision\left(Prec\right)=\frac{tp}{tp+fp}$$

(3) $$Recall\left(Rec\right)=\frac{tp}{tp+fn}$$

(4) $$F_{measure}\left(F_{1}\right)=\frac{2\times precision\times recall}{precision+recall}$$

A set of 500 images from each class is used to compute the confusion matrix parameter (tp (true positives), fp (false positives), tn (true negatives) and fn (false negatives)), and these images were not used for training the image classifier. Table 3 gives the statistical measures results computed through the confusion matrix parameter.

The offline test results show that all the six classification frameworks show an accuracy above 90%. Among the classes, ash, soil, sand, and hair showed the best classification accuracy, since the images were visually distinct in color and texture. The lowest classification accuracy was reported for the class paper and paint, since a scrap of paint and paper bit possesses almost the same visual features under 10× magnification by the camera. The darknet53 model showed the lowest classification accuracy for the class paper with 66.81%. On the other hand, the no-dirt class representing the samples devoid of dirt, which is a critical factor in determining the surface’s cleanliness, showed high accuracy in all trained models. Even though the evaluation is performed on GPU, the ResNet101, Darknet53, and ResNet50 reported comparatively lower inference time than VGG-16, VGG-11, and MobileNetV2. The difference in inference time is attributed to the number of operations within the network. Regarding ResNet50, ResNet101, and Darknet53, there are 23 M, 44.5 M, and 40.5 M parameters, respectively. Whereas in the case of VGG-11, VGG-16, and MobilenetV2, there were 133 M, 138 M, and 3.4 M parameters trained, respectively. The statistical measurements reported in the offline test results show that the dataset gathered for dirt classification is un-biased between the classes and it can be used alongside the standard deep-learning models.

### 4.5. Real-Time Robot-Aided Cleaning Inspection

**Figure 7:**
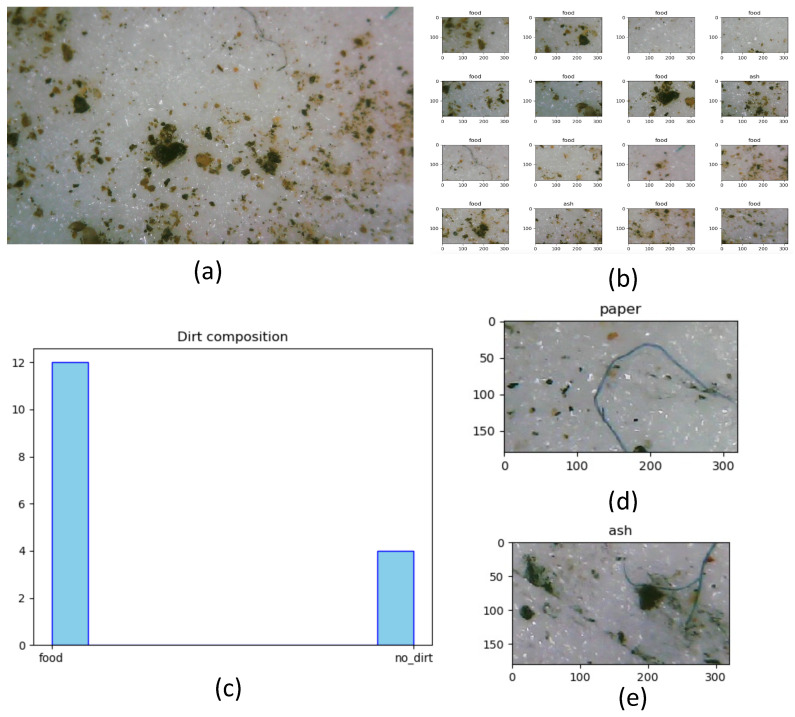
The test images collected in real time using the BELUGA robot (**a**), classified images (**b**), histogram of classified dirts from single sample (**c**), an exmple for wrongly classified image (**d**), and overlapping of dirt specks (**e**).

In addition to the offline test, the usability of the curated dataset is analyzed in a real-time cleaning inspection use case with the BELUGA robot. We have chosen an environment for testing which is similar to the chosen regions for data collection. DarkNet53 was used for rolling out the real-time inference considering the best performance in k-fold cross-validation and offline testing. Since the BELUGA robot’s embedded computer is devoid of GPU, the inference is completed by establishing a communication with a remote server running with GPU. Using the BELUGA robot, the five dirt samples are collected each from the food court, walkway and indoor office space. For every sample image captured by the digital microscope after dust lifting, the sample image is divided into 16 images, matching the training dataset. The models loaded with weights were trained, and the classification of dirt is completed for every 16 images from the collected sample. Figure 7 shows the images classified from the dirt sample and the estimated dirt composition.

#### Comparison with Offline Test Results

Out of the 15 sample images, which were divided into 240 test image samples, 203 samples reported the right classification with an admissible accuracy of 84.58%. The model’s real-time accuracy was less than the offline test results. Despite the above-mentioned shortcomings, the model trained with the curated dataset showed a good accuracy, which is acceptable for the dirt composition estimation in cleaning auditing.

## 5. Discussion

The experiments results conducted showed the prepared dataset’s usability on popular deep learning models such as VGG-16, VGG-11, MobileNet V2, ResNet50, ResNet101, and Darknet53. Upon rolling out the trained with the prepared dataset for real-time inference, an admissible accuracy was observed. During the course of our dataset curation, certain limitations identified include the narrow field of view of the camera and the overlapping of multiple dirt classes in the sample image. Moreover, certain dirt particles share similar textures, making them difficult to be distinguished. Although the data inference is substantially faster, the dirt data collection is found to be slow, since it involves adhesive dirt lifting. Unlike the offline test results, false-positive occurrences are reported in the real-time test results (shown in Figure 7d), which are contributed to the following factors:

- Overlapping of multiple specks of dirt classes in a sample image (shown in Figure 7e);
- The shaking of adhesive tape during the actuation of the sensor may result in blur images that eventually lead to a wrong classification;
- Encountered dirt specks with very close visual resemblance make it indistinguishable for the model to classify.

## 6. Conclusions and Future Works

A dirt image dataset was proposed for AI-based dirt classification for automated cleaning auditing. Our in-house developed cleaning inspection robot BELUGA was used to gather the dirt sample images from a semi-indoor environment. We identified nine visually distinct dirt classes, and 3000 10× magnified microscope images for each class are gathered for the dataset. The usability of the collected dirt dataset was evaluated by analyzing the training and evaluation accuracy in six state-of-the-art image classifier models. The k-fold cross-validation method with a cross-entropy loss function was used to compute the model’s training accuracy, and the statistical measure function was used to assess the classification accuracy of models trained using our dirt image dataset. During the training, a minimal standard deviation for training accuracy for every k-fold cross-validation iteration is observed, which indicates the unbiased nature of the collected dataset. The offline test results indicate that all the trained models scored above 90% accuracy for all classes. The quality of the dataset is further validated by rolling out the trained dataset for real-time cleaning auditing using an in-house developed BELUGA robot. The accuracy of real-time testing was comparatively less compared to the offline test results, which is mainly attributed to the overlapping of multiple specks in the same region of the sample image. The motion blur was also introduced in the dirt lifting process, which diminished the accuracy of real-time dirt analysis. In addition, the time taken for overall dirt sample collection was slower because the adhesive dirt-lifting process was time-consuming. Our future research will be focused on the following areas:

- Combining microbial and chemical analysis in the process of sample auditing;
- Incorporating novel autonomous algorithms toward dirt exploration;
- A comprehensive study comparing the different algorithms with respect to cleaning auditing;
- Exploring the usability of the current dataset for instance segmentation of dirt particles;
- Improving the current dataset by expanding the number of dirt classes and open-sourcing the dataset.

## Figures and Tables

**Table 1:** The classes of identified domestic dirt.

Class (Number)	Sample Images				
Ash	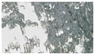	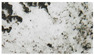	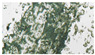	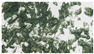	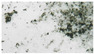
Hair	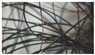	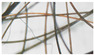	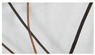	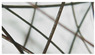	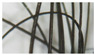
Sand	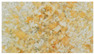	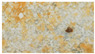	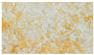	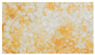	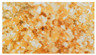
Soil	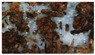	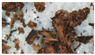	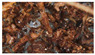	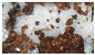	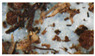
Paper	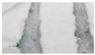	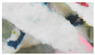	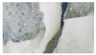	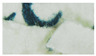	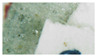
Paint	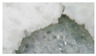	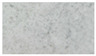	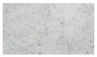	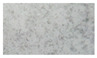	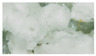
Food	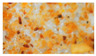	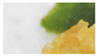	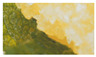	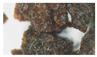	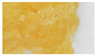
Fibre	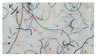	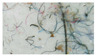	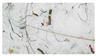	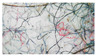	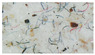
No-dirt	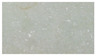	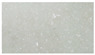	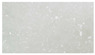	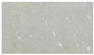	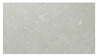

**Table 2:** Statistical measures for dirt classification.

Model	Average Accuracy (%)	K-Fold Standard Deviation
VGG-11	94.77	5.82
VGG-16	92.77	3.90
MobileNetV2	94.65	3.44
ResNet50	96.58	2.05
ResNet101	96.54	1.89
Darknet53	95.801	3.06

**Table 3:** Statistical measures for dirt classification.

Model	Class	Measurements			
		Precision	Recall	F1	Accuracy %
VGG-16	Ash	0.9803	0.9970	0.9886	99.70
	Food	0.9918	0.9675	0.9795	96.65
	Fibre	0.9559	0.9644	0.9601	96.44
	Paper	0.8621	0.9336	0.8964	89.64
	Paint	0.9689	0.9291	0.9485	92.91
	Soil	0.9993	0.9970	0.9981	99.70
	Sand	0.9878	0.9943	0.9910	99.43
	Hair	0.9897	0.9998	0.9948	99.99
	No-dirt	0.9971	0.9941	0.9956	99.41
VGG-11	Ash	0.9900	0.9960	0.9930	99.60
	Food	0.9709	0.9787	0.9748	97.87
	Fibre	0.9847	0.9555	0.9699	95.55
	Paper	0.8482	0.9458	0.8944	94.58
	Paint	0.9914	0.9110	0.9495	91.10
	Soil	0.9983	0.9985	0.9982	99.85
	Sand	0.9906	0.9820	0.9863	98.20
	Hair	0.9622	0.9977	0.9796	99.77
	No-dirt	0.9798	0.9985	0.9891	99.85
MobileNet V2	Ash	0.9950	0.9920	0.9935	99.20
	Food	0.9907	0.9638	0.9770	96.38
	Fibre	0.9700	0.9585	0.9642	95.85
	Paper	0.9108	0.9038	0.9073	90.38
	Paint	0.9707	0.9516	0.9610	95.16
	Soil	0.9963	0.9993	0.9978	99.93
	Sand	0.9843	0.9970	0.9906	99.70
	Hair	0.9589	0.9954	0.9768	99.54
	No-dirt	0.9812	0.9956	0.9883	99.56
ResNet50	Ash	0.9930	0.9880	0.9905	99.30
	Food	0.9956	0.9606	0.9778	96.06
	Fibre	0.9761	0.9703	0.9732	97.03
	Paper	0.9454	0.9392	0.9423	93.92
	Paint	0.9660	0.9758	0.9709	97.58
	Soil	0.9991	0.9985	0.9993	99.85
	Sand	0.9825	0.9973	0.9899	99.73
	Hair	0.9852	0.9965	0.9908	99.65
	No-dirt	0.9963	0.9985	0.9974	99.85
ResNet101	Ash	0.9990	0.9970	0.9980	99.70
	Food	0.9919	0.9755	0.9836	97.55
	Fibre	0.9910	0.9822	0.9866	98.22
	Paper	0.9016	0.9624	0.9310	96.24
	Paint	0.9889	0.9499	0.9690	94.99
	Soil	0.9997	0.9985	0.9993	99.85
	Sand	0.9899	0.9970	0.9934	99.70
	Hair	0.9730	0.9965	0.9846	99.65
	No-dirt	0.9971	0.9985	0.9978	99.85
Darknet53	Ash	0.9979	0.9699	0.9837	96.99
	Food	0.9382	0.9307	0.9344	93.07
	Fibre	0.9939	0.9614	0.9774	93.07
	Paper	0.9001	0.6681	0.7570	66.81
	Paint	0.9861	0.9583	0.9720	95.83
	Soil	0.9901	0.9993	0.9996	99.93
	Sand	0.9568	0.9111	0.9334	91.11
	Hair	0.6506	0.9965	0.7872	99.65
	No-dirt	0.9728	0.9985	0.9855	99.65

## Data Availability

Not applicable.
